# Multiple RNA-binding proteins associated with long interspersed element-1 encoded ORF1p are targeted by the autoimmune response in systemic lupus erythematosus

**DOI:** 10.46439/immunol.2.022

**Published:** 2023

**Authors:** Kennedy C. Ukadike, Alyssa N. Colyer, Bhargavi Duvvuri, Anders A. Bengtsson, Martin S. Taylor, John LaCava, Christian Lood, Tomas Mustelin

**Affiliations:** 1 Division of Rheumatology, Department of Medicine, University of Washington School of Medicine, Seattle, WA, USA; 2 Current address: Renown Rheumatology, Renown Health and Department of Internal Medicine, University of Nevada Reno School of Medicine, Reno, NV, USA; 3 Division of Rheumatology, Lund University and Skåne University Hospital, Lund, Sweden; 4 Massachusetts General Hospital, Boston, MA, and Whitehead Institute, Cambridge, MA, USA; 5 The Rockefeller University, New York, NY, and European Research Institute for the Biology of Ageing, University Medical Center Groningen, Groningen, The Netherlands

**Keywords:** Systemic lupus erythematosus, Autoantibodies, ORF1p, LINE-1, RO60, MOV10, Arthritis, Type I interferon

## Abstract

Systemic lupus erythematosus (SLE) is a relatively common autoimmune disease characterized by the presence of autoantibodies against nucleic acids and proteins that associate with them, such as the ORF1p protein encoded by the long interspersed element-1 (LINE-1 or L1). Because well-known lupus autoantigens like RO60 associate with ORF1p in macromolecular assemblies, together with many other RNA-binding proteins, we tested whether these other proteins are also recognized by IgG autoantibodies in SLE patients. By ELISAs and immunoblots, we detected autoantibodies in the serum of SLE patients recognizing proteins encoded by *LARP7*, *MOV10*, *ZCCHC3*, *MEPCE*, *YARS2*, *RPL18A*, *RPL27A*, and *H2BC17* (p<0.05), but not *CORO1B*, *DDX6*, *PABPC1*, and *PABPC4*, and were mostly absent or low in healthy controls. The titers of antibodies against RO60, LARP7, MOV10, and MEPCE were higher (p<0.05) in those patients who also had anti-ORF1p autoantibodies. These antibodies also correlated with dsDNA antibodies, the presence of arthritis, and higher levels of type I interferons. A cluster analysis revealed that all these autoantibodies collectively identified patients with more active disease. We conclude that patients with SLE have elevated IgG autoantibodies not only against the L1-encoded ORF1p, but also against 8 other proteins that co-localize with ORF1p in RNA-rich granules. These autoantibodies are higher in patients who have autoantibodies to ORF1p and together correlate with elevated type I interferon levels. Our findings are compatible with the notion that ORF1p-containing ribonucleoprotein granules are a target of the autoimmunity in SLE.

## Graphical Abstract

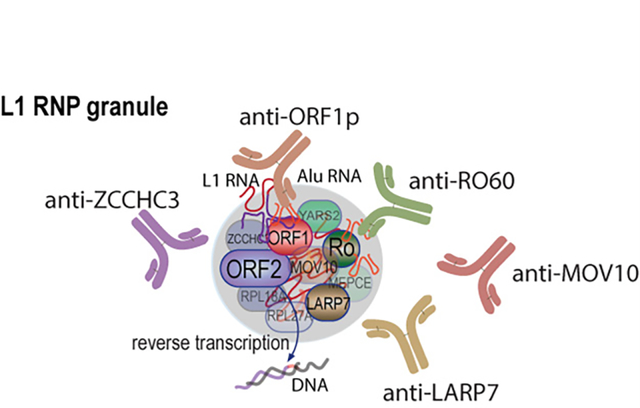

## Introduction

Type I interferons (IFN) are elevated in the blood of most patients with systemic lupus erythematosus (SLE) [1–4], dermatomyositis [5], and Sjögren’s syndrome [6]. Since type I IFNs are best known for their central role in anti-viral immunity [7], and are produced as a consequence of the recognition of viral DNA or RNA, many investigators suspect that pathogenic nucleic acids are also present in SLE [8]. In support of this notion, a subset of SLE patients with active SLE were reported to have elevated cyclic guanosine adenosine phosphate (cGAMP) [9], which is exclusively synthesized by the DNA-sensor cGAS when it binds non-nucleosomal DNA. A subset of SLE patients also have oligomerized mitochondrial antiviral signaling (MAVS) protein [10] as a result of the binding of non-self RNA by the RNA sensors MDA5 and/or RIG-I [11–14]. The sources of nucleic acids that trigger cGAS, MDA5, and RIG-I are unknown [8], but double-stranded RNA has been shown to trigger type I IFN production through Z-DNA binding protein-1 (ZBP1) [15], which recognizes both RNA and DNA in the Z-configuration, in Crohn’s disease [16] and in several autoinflammatory disease models [17]. Interestingly, the most of the identified double-stranded RNA species were derived from retrotransposon sequences [16,17]. One family of such sequences is the autonomous retrotransposon long interspersed element-1 (LINE-1 or L1) which constitutes nearly 20% of the human genome [18–21]. Intact copies of L1 encode two proteins, the 40-kDa RNA-binding protein ORF1p and the 149-kDa endonuclease and reverse transcriptase ORF2p. The latter can synthesize DNA that triggers DNA sensors, such as cGAS, leading to type 1 IFN production [22].

We recently reported that sera from SLE patients contain high levels of IgG autoantibodies against ORF1p [23]. The titers of these autoantibodies were higher in patients during exacerbations than during periods of low disease activity and they correlated with type I IFNs, SLEDAI, anti-dsDNA, nephritis, and complement consumption. In contrast, patients with scleroderma or rheumatoid arthritis did not have anti-ORF1p autoantibodies [23]. We also found that pediatric SLE patients have IgA autoantibodies, in addition to IgG, against ORF1p [24]. Children with dermatomyositis and juvenile idiopathic arthritis had marginally elevated anti-ORF1p autoantibodies. In pediatric SLE patients, these autoantibodies correlated with disease activity and serological measures of the disease [24]. They also correlated with circulating neutrophil-derived cell-free DNA, indicative of elevated non-apoptotic neutrophil death [25], which may be related to our detection of ORF1p in SLE neutrophils. Transcripts derived from L1, as well as the encoded proteins, have also been reported to be elevated in the kidneys of SLE patients and in the salivary glands of patients with Sjögren’s syndrome [26,27].

A substantial quantity of ORF1p accumulates in cytosolic, RNA-containing macromolecular assemblies. In different studies, these have been characterized as bearing resemblance to different RNA granule-types (e.g., stress granules, p-bodies, or insulin like growth factor 2 mRNA binding protein 1 granules) [28,29] leaving open the possibility that L1 granules may be distinct entities. Nevertheless, ORF1p-containing cytoplasmic granules also contain several other RNA-binding proteins [30–33], including the well-known SLE autoantigen RO60 [32] and La-related protein 7 (LARP7) [34]. We therefore asked whether these other ORF1p-associated proteins are also targeted by the autoimmune response in SLE, as suggested by our previous observation that patient sera reacted with at least seven unidentified proteins in purified L1 RNA particles [23]. Here we report that 8 of 13 tested ORF1p-interacting proteins produced recombinantly are recognized better by patient than healthy control IgG in a statistically significant manner. While each patient had a unique profile of such antibodies, there was a general correlation between their titers and those of anti-ORF1p antibodies, as well as SLE disease activity, and type I interferons. Besides anti-RO60 antibodies, high titers of autoantibodies recognizing Moloney leukemia virus 10 (MOV10) and Zinc finger CCHC domain-containing 3 (ZCCHC3) were notable.

## Materials and Methods

### SLE patients

Serum samples from a cohort of SLE patients with low activity disease (n=32; designated as ‘low’ in Figures), defined as having a SLE Disease Activity Index (SLEDAI) ≤ 4, and samples from the same patients when their disease activity was higher, SLEDAI ≥ 6 (n=2l; ‘high’), and healthy individuals (n=20) were recruited at the Department of Medicine, Skåne University Hospital, Lund, Sweden. This patient cohort has been described in great detail previously [35–37], including their type I interferon activity, which was measured with a bioassay. The study was approved by Lund University local ethics board (LU06014520, and LU 378–02). Informed written consent was obtained from all participants according to the Declaration of Helsinki.

### Recombinant proteins

Thirteen proteins known to associate with ORF1p were expressed as glutathione S-transferase fusion proteins and purified as described [38] by CDI Laboratories (https://cdi.bio). A Coomassie Blue stained gel revealed that the proteins had minimal contaminating yeast proteins, but in some cases smaller proteins likely representing partial proteolysis. Recombinant ORF1p was as described before [23].

### Enzyme-linked immunosorbent assays (ELISAs)

Purified proteins were adsorbed onto 96-well polystyrene plates at 50 ng/well in 1X phosphate buffered saline (PBS) buffer, pH 7.4, overnight at 4°C, washed in PBS with 0.05% (v/v) Tween-20, and blocked in 2% (w/v) bovine serum albumin (BSA) in PBS overnight at 4°C. The protein-coated wells were washed again before patient, or healthy control, sera were added at 1:200 dilution in 1% (w/v) BSA in PBS for a 2 h incubation at room temperature. The primary reaction was washed extensively and then incubated for 2 h with 1:2000 dilution of horseradish peroxidase-conjugated goat anti-human IgG. The secondary reaction was washed as before, developed with TMB substrate, with the color reaction terminated with 2N sulfuric acid, and the absorbance measured at 450 nm using BioTek Gen5 Microplate Reader.

### Immunoblotting

1 μg of each protein per lane was resolved by sodium dodecyl sulfate (SDS)-polyacrylamide gel electrophoresis (PAGE) and transferred to a nitrocellulose membrane, followed by blocking in SuperBlock Buffer (from ThermoFisher Scientific). The first step of protein detection was by immunoreacting with 1:100 diluted patient, or healthy control, serum for 2 h at room temperature. Following extensive washes with tris buffered saline with 0.05% Tween-20 (TBST), the secondary detection reaction was performed by incubating with 1:2000 dilution of horse radish peroxidase-conjugated goat anti-human IgG for 1 h at room temperature. After several washes, the protein bands were detected by reacting with chemiluminescence substrate followed by visualization with BioRad Image Lab machine software.

### Cluster analysis

Hierarchical clustering on 53 SLE patients (active n=21; inactive n=32) was performed using Euclidean distance metric and Complete-linkage clustering method using the R v4.0.2 pheatmap v1.0.12 (https://www.r-pkg.org/pkg/pheatmap).

### Statistics

For non-paired sample sets with non-Gaussian distribution, Mann-Whitney U test, and Spearman’s correlation test were used. For paired sample sets, Wilcoxon matched-pairs signed rank test was used. In some analyses, logistic regression analysis was used for dichotomized variables. The 95^th^ percentile of the healthy control data set was used as cut-off for antibody positivity in the corresponding SLE data sets. GraphPad Prism 9.4.1 and IBM SPSS were used for the analyses. A p-value of <0.05 was considered statistically significant.

## Results

### IgG autoantibodies against 9 of 13 L1 ORF1p-associated proteins

Thirteen proteins known to interact with ORF1p [28,33,39], encoded by *RO60*, *LARP7*, *MOV10*, *ZCCHC3*, *MEPCE*, *YARS2*, *RPL18A*, *RPL27A*, *H2BC17*, *CORO1B*, *DDX6*, *PABPC1*, *PABPC4*, were expressed in yeast, purified, and used to perform ELISAs. All ELISA results were normalized using a selected high-titer patient included as a titration curve standard on every 96-well ELISA plate.

**Figure 1.**
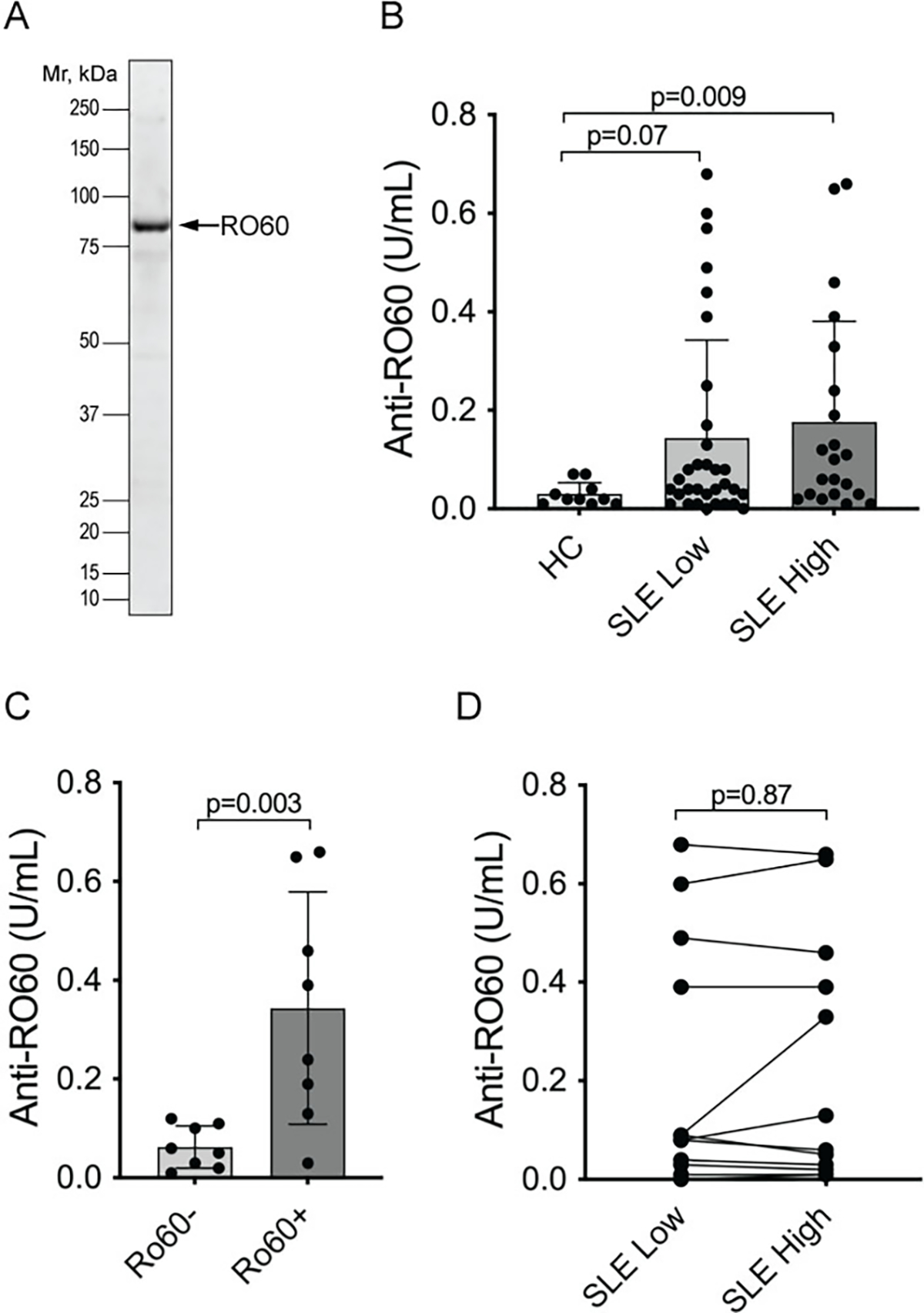
Autoantibodies react with RO60 in SLE patients. **A.** Coomassie stain of the used recombinant GST-RO60 protein. **B.** ELISA with serum from healthy controls (HC), SLE patients when their SLEDAI was ≤ 4 (SLE low) and the same patients at another time point when their disease was more active with SLEDAI ≥ 6 (SLE high). Statistical significance by Mann-Whitney U test. **C.** ELISA results in the SLE high set segregated by having a negative (Ro60−) or positive (Ro60+) clinical laboratory test for anti-RO60 antibodies (n=16). Statistical significance by Mann-Whitney U test. **D.** Connecting the individual SLE patients’ two values in panel B (n=12) to illustrate the lack of significant change between active and inactive disease. Statistical significance was calculated using Wilcoxon matched-pairs signed rank test.

Autoantibodies against RO60 are well documented [40] and were considered a positive control in our experiments. Indeed, 47% of the SLE patients were positive, *i.e.,* above the 95^th^ percentile of normal (Figure 1B), while the healthy controls all had very low values. Furthermore, our ELISA data were very similar to clinical laboratory measures of anti-RO60 autoantibodies: very low in those patients who had previously tested negative for anti-RO60 and covering a range of values in those who had been positive (Figure 1C). Only one previously positive patient had a very low value in our ELISAs. As reported before, the titers were similar in samples taken from the SLE patients at a time when the disease was active, and a sample taken when the disease activity was low (Figure 1D).

**Figure 2.**
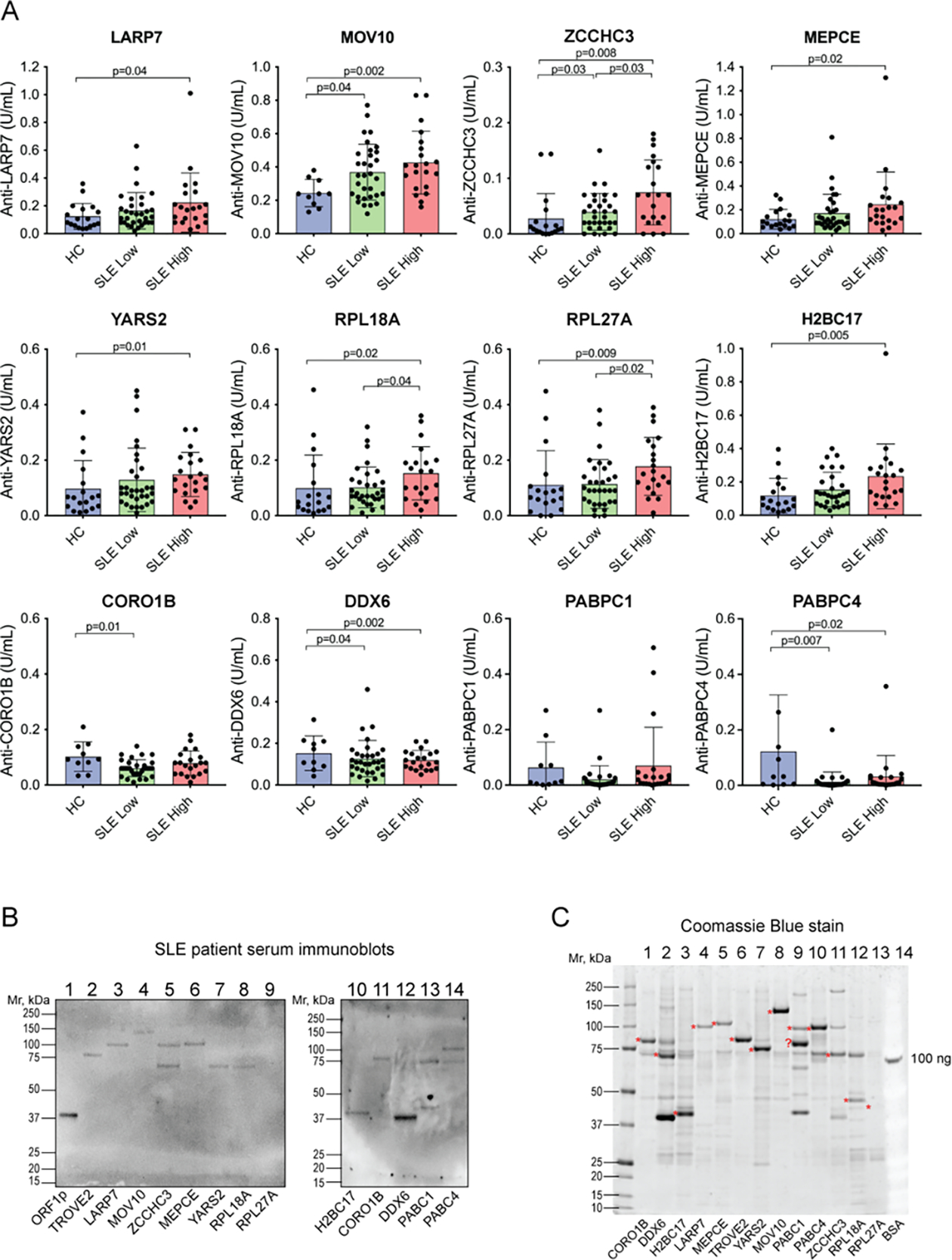
ELISAs for IgG autoantibodies reactive against 12 proteins known to be associated with L1 ORF1p. **A.** Quantitation by ELISA of IgG bound to the indicated proteins. Statistical significance was calculated by the Mann-Whitney U test; n.s., not significant. Note that CORO1B, DDX6, PABPC1, and PABPC4 did not yield increased reactivity in SLE. **B.** Immunoblot of ORF1p and the same 12 proteins (1 μg) with serum from a patient with high reactivity against ORF1p and the ELISAs in panel A. **C.** Coomassie stain of the 12 proteins, plus 100 ng bovine serum albumin (BSA) as a loading control. The red asterisks denote the expected size of the respective GST-fusion protein.

ELISAs with the 12 other proteins gave a range of outcomes. A statistically significant increased reactivity in SLE over healthy controls was seen against LARP7, MOV10, ZCCHC3, Methylphosphate Capping Enzyme (MEPCE), Tyrosyl-tRNA Synthetase 2 (YARS2), Ribosomal Protein L18a (RPL18A), Ribosomal Protein L27a (RPL27A), H2B Clustered Histone 17 (H2BC17), but not against Coronin 1B (CORO1B), DEAD-box Helicase 6 (DDX6), Poly(A) Binding Protein Cytoplasmic 1 (PABPC1), and Poly(A) Binding Protein Cytoplasmic 4 (PABPC4) (Figure 2A). However, there were large differences in pattern between these proteins: the IgG autoantibodies against some were elevated in a statistically significant manner in both inactive and active disease (MOV10 and ZCCHC3), while others were only statistically significant in patients with higher disease activity (LARP7, MEPCE, YARS2, RPL18A, RPL27A, and H2BC17). Furthermore, some autoantibodies were positive, defined as higher than the 95^th^ percentile of the healthy donors, in only a minority of patients (*e.g*. 5.7% for YARS2 and 11.3% for LARP7 and MEPCE), while for other proteins nearly half of the patients were positive, *e.g.* 47% for RO60 and MOV10. Autoantibodies against MOV10 and ZCCHC3 also had higher titers in patients with active disease versus those with low activity disease. It should also be noted that some proteins were recognized by IgG antibodies in a number of healthy individuals, including YARS2, RPL18A, RPL27A, CORO1B, PABPC1, and PABPC4. For all these reasons, we considered MOV10 and ZCCHC3 to be of the highest interest. Most of the proteins were also recognized by patient IgG in immunoblots (Figure 2B), which confirmed that the correct proteins were recognized (as opposed to minor contaminants from yeast expression; Figure 2C).

### Autoantibodies correlate with anti-ORF1p autoantibodies

**Figure 3.**
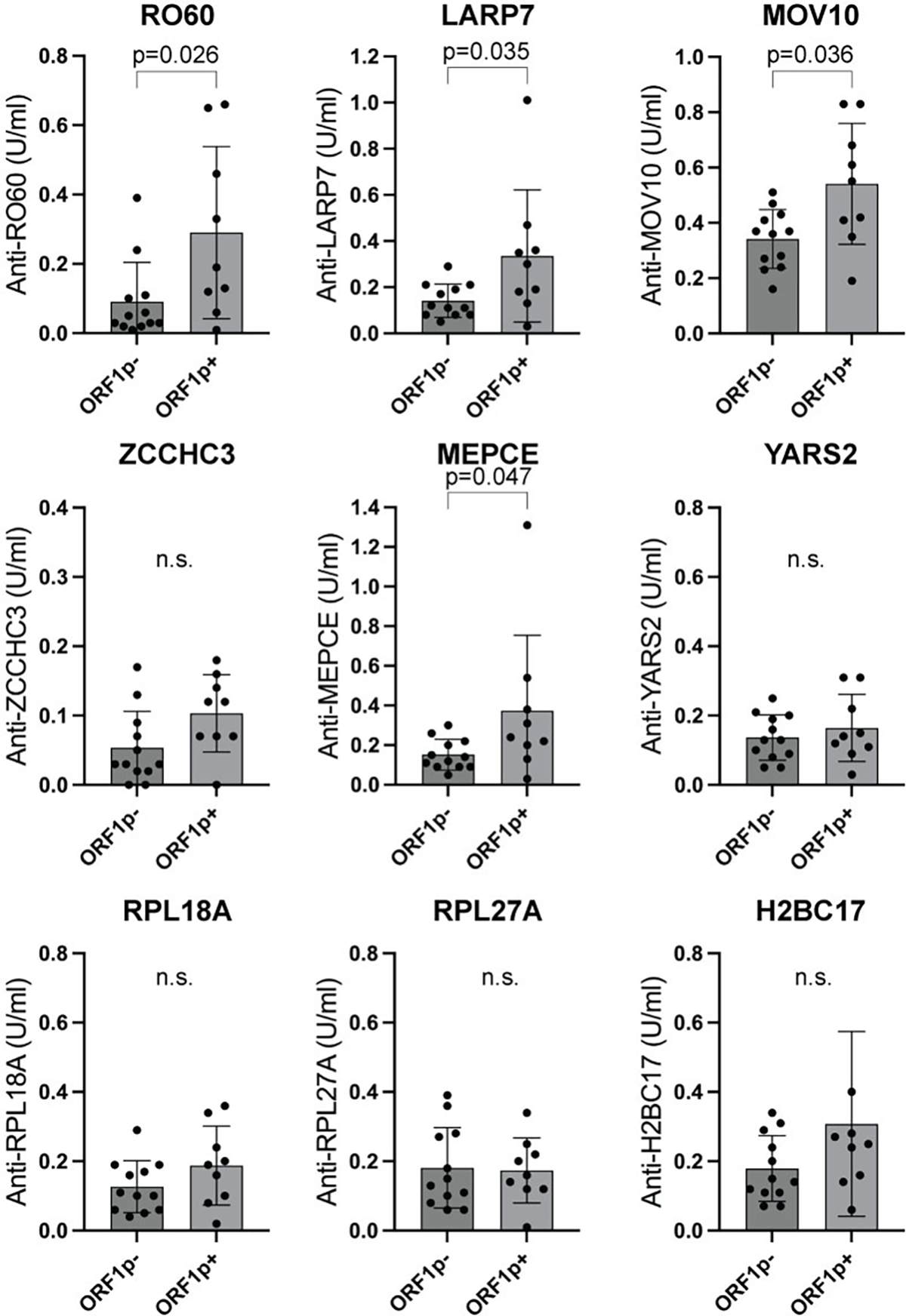
Autoantibodies against 9 of the ORF1p-associated proteins are mainly present in patients with anti-ORF1p autoantibodies. Titers of IgG antibodies to the indicated proteins in patients without (ORF1−) or with (ORF1p+) anti-ORF1p antibodies. Statistical significance was calculated using the Mann-Whitney U test; n.s., not significant. The 3 other proteins did not correlate with the anti-ORF1p antibodies.

Next, we asked whether the presence of these autoantibodies relates to the titers of anti-ORF1p antibodies in the same patients. If the proteins are released from dying cells as a macromolecular aggregate that also contains nucleic acids, one would expect the humoral autoimmune response against all these proteins to be linked. The patients with active SLE were divided into two groups based on their anti-ORF1p titers (low versus high), and the titers of the autoantibodies against the other proteins compared between the two groups. As shown in Figure 3, the autoantibodies against RO60, LARP7, MOV10, MEPCE were clearly higher in the anti-ORF1p-high patients. Anti-ZCCHC3 autoantibodies also showed a similar trend, which barely missed statistical significance. The other proteins did not correlate with anti-ORF1p status.

**Figure 4.**
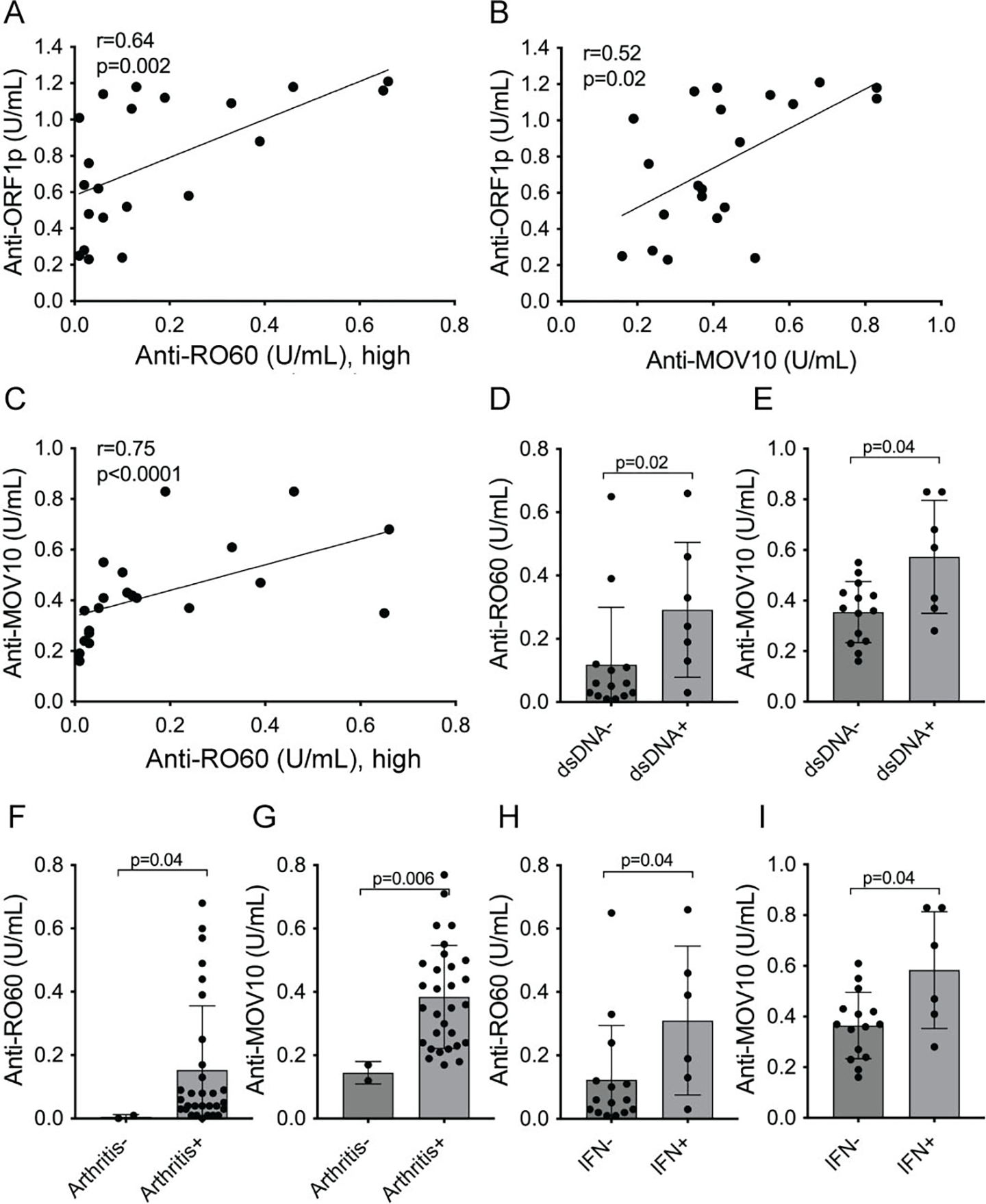
Association of anti-MOV10 and anti-RO60 with anti-ORF1p and patient parameters. **A.** linear regression analysis for correlation between anti-ORF1p and anti-RO60 values for each patient with active SLE (n=21). **B.** similar analysis for anti-ORF1p versus anti-MOV10 (n=21). **C.** similar analysis for anti-MOV10 versus anti-RO60. **D.** anti-RO60 titers in SLE high patients segregated into those without (dsDNA−) or with (dsDNA+) a positive clinical laboratory test in the medical records for anti-dsDNA. **E.** same for anti-MOV10. **F.** anti-RO60 titers in the SLE low population segregated by the absence (Arthritis−) or presence (Arthritis+) of joint pain and swelling. **G.** same for anti-MOV10. **H,** anti-RO60 titers in SLE patients segregated into those without (IFN−) or with (IFN+) signal in the serum IFN bioassay. **I.** same for anti-MOV10.

The correlation between anti-RO60 and anti-MOV10 versus anti-ORF1p titers were also tested as continuous variables. As shown in Figure 4, anti-RO60 correlated with anti-ORF1p in a statistically significant manner (r=0.64, p=0.002; Figure 4A), as did anti-MOV10 (r=0.52, p=0.02; Figure 4B). Similarly, anti-MOV10 and anti-RO60 antibodies also correlated with each other (r=0.75, p<0.0001; Figure 4C).

### Associations of anti-MOV10 autoantibodies with clinical parameters and type I IFN

Similar to anti-RO60, anti-MOV10 were higher in SLE patients with anti-dsDNA antibodies (Figures 4D and 4E), in those with arthritis (Figures 4F and 4G), and in those with elevated type I interferons (Figures 4H and 4I). Autoantibodies against the other ORF1p-associated proteins showed trends towards similar correlations with clinical or laboratory parameters but did not reach statistical significance.

### Patterns of autoantibodies by cluster analysis

**Figure 5.**
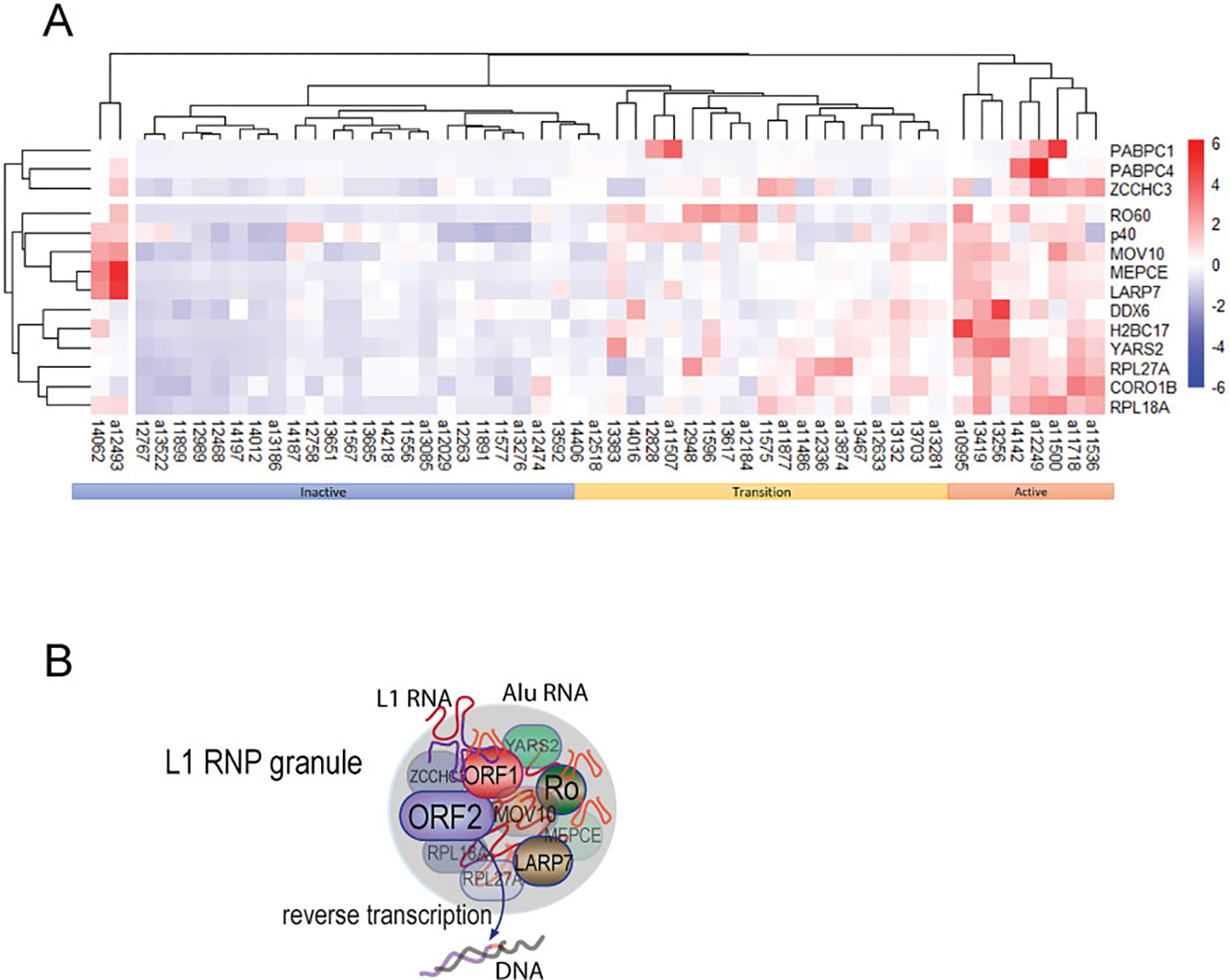
Summary of the set of autoantibodies against ORF1p-associated proteins. **A.** Heatmap analysis of the antibodies showing a segregation by disease activity. Patients with low disease activity and high disease activity are indicated, while those in the intermediate clade are renamed ‘intermediate’. ‘p40’ refers to ORF1p. **B.** Schematic illustration of the macromolecular aggregate of ORF1p and the proteins associated with it, including ORF2p.

Clustering analysis (Figure 5A) of the new autoantibodies and anti-ORF1p in the same patients [23], together with disease activity, revealed three major clusters, one enriched for patients (n=8) with active disease where patients were positive for most of the autoantibodies. A second cluster, labeled inactive, contained patients (n=23) that were negative for most autoantibodies. A third cluster was characterized by intermediate levels of antibodies and mixed patients (n=20). We refer to them as ‘transition’ in Figure 5, hypothesizing that they represent a transition between inactive and active disease based on the pattern of autoantibody levels. There was considerable heterogeneity within these patients *visa-vi* individual autoantibodies, precluding the detection of this transition through analysis of any individual antibody. Lastly, two outliers with inactive disease, but high autoantibodies, were also noted.

## Discussion

Our results reveal that SLE patients have IgG autoantibodies against several proteins that co-localize with the L1-encoded ORF1p retrotransposon protein in the RNA-rich cytosolic macromolecular assemblies (Figure 5B). We show that proteins encoded by *RO60*, *LARP7*, *MOV10*, *ZCCHC3*, *MEPCE*, *YARS2*, *RPL18A*, *RP27A*, *H2BC17*, but not *CORO1B*, *DDX6*, *PABC1*, and *PABC4*, are indeed targeted by autoantibodies in SLE patients in a manner that aligns with the presence of anti-ORF1p autoantibodies. Because patients with low anti-ORF1p antibodies have low levels of autoantibodies against all these other proteins and none of them correlate as distinctly with clinical measures and laboratory values associated with active SLE, we speculate that ORF1p plays a more prominent role in provoking an immune response and that the other proteins may become autoantigens as a consequence; they may represent ‘innocent bystanders’ for which various degrees of central tolerance exists, but become targeted by ‘epitope spreading’ boosted by the adjuvant effect of TLR7-stimulating RNA present in the ORF1p-containing complexes. Alternatively, some of these proteins may also be immunogenic in their own right. Most likely would be MOV10, the autoantibodies against which show correlations to patient parameters similar to those of anti-ORF1p [23], but, interestingly, differ by correlating positively with arthritis. Anti-MOV10 titers also did not correlate with SLE nephritis, while anti-ORF1p titers did [23], but instead inversely correlated with photosensitivity (not shown). As yet, we have no mechanistic explanations for these differential correlations with clinical manifestations of SLE, but the known functions of these proteins provide some basis for speculation. The patients’ major histocompatibility complex (HC) haplotype may also play a role.

MOV10 is an interferon-inducible 110-kDa RNA helicase with an important role in the defense against viruses, including influenza [41], hepatitis B [42], and human immunodeficiency virus [43]. In the case of hepatitis B, MOV10 prevents reverse transcription [42] of the viral RNA genome into the circular cDNA that maintains a chronic infection. MOV10 also counteracts the reverse-transcription dependent retrotransposition of L1 elements [44,45]. The knockout of *mov10* in mice is lethal [46], suggesting that its functions are critically important beyond viral defense.

ZCCHC3 is also an interferon-inducible gene with a role in innate defense against viruses. It has been reported to act as a co-sensor for pathogenic DNA together with cGAS [47] and to promote the sensing of pathogenic RNA by RIG-I [48] and dsRNA by TLR3 [49]. Its presence in ORF1p-containing complexes rich in RNA may facilitate the detection of abnormal RNA species (such as Alu RNAs that co-assemble in L1 ribonucleoprotein granules) and/or extrachromosomal DNA generated by L1 reverse transcriptase, ORF2p, in the cytoplasm or nucleus [22].

Collectively, our data are compatible with the proposition that autoantigens are ‘linked’ because they are presented to the adaptive immune system as protein-RNA complexes that contain more immunogenic components that bring along less immunogenic ones through epitope spreading to become the targets of autoantibodies of various affinities and titers. We propose that L1-containing macromolecular assemblies (Figure 5B) escape dying cells and that they become targets for autoimmunity because some of the proteins, like ORF1p, are more immunogenic and because the RNA in these assemblies acts as an adjuvant to stimulate antigen presentation and the activation of autoreactive B cells through TLR stimulation. The existence of such protein-RNA complexes has been demonstrated by the sequencing of RNA in complex with circulating RO60, which contained L1 and Alu RNA [32]. These protein-RNA aggregates likely also contained ORF1p, which forms stable trimers with high affinity for RNA, including its cognate mRNA as well as Alu RNA. Based on the composition of purified ORF1p/RNA assemblies [30–33], the other proteins studied in this paper are likely also present in the same circulating particles.

We recently found that ORF1p protein is detectable in a subset of circulating neutrophils both in pediatric and in adult SLE patients, particularly in those with active disease [24]. The percentage of positive neutrophils correlated with disease activity measured by SLEDAI. The positive neutrophils were both regular higher-density neutrophils, as well as the lower-density granulocytes, which are increased in lupus [50]. We also found that measures of neutrophil activation and death were significantly elevated in the blood of lupus patients with active disease. Because neutrophils are abundant and appear to die by a non-apoptotic mechanism (either NET formation, necroptosis, or ferroptosis), they may well release ORF1p/RNA particles containing many of the proteins included in the present study. If these complexes are indeed the main presentation of the autoantigens that give rise to autoantibodies recognizing ORF1p, as well as RO60, LARP7, MOV10, ZCCH3, MEPCE, YARS2, RPL18A, RPL27A, and H2BC17, then it is not surprising that they seem to co-correlate. In this scenario, the autoantibodies are ‘linked’ because the autoantigens are physically linked to particles that are released from cells during periods of active disease. This release will boost existing memory B cells to renew their production of the cognate autoantibodies, explaining why they tend to be higher in patients with active disease. Other large protein-RNA complexes or organelles may also be released from dying cells, each giving rise to a set of linked autoantigens, such as those targeting the Argonaute proteins (AGO1, AGO2, and AGO3), major vault protein (MVP), and the ribosome (RPLP2) [51]. Exploring these connections may lead to important insights into the mechanisms of how autoantibodies arise and why certain proteins became targets more frequently than others.

## Data Availability

All data will be made available for research purposes upon request.
